# Preschoolers Favor Their Ingroup When Resources Are Limited

**DOI:** 10.3389/fpsyg.2018.01752

**Published:** 2018-09-19

**Authors:** Kristy Jia Jin Lee, Gianluca Esposito, Peipei Setoh

**Affiliations:** ^1^Psychology, School of Social Sciences, Nanyang Technological University, Singapore, Singapore; ^2^Department of Psychology and Cognitive Science, University of Trento, Trento, Italy

**Keywords:** fairness, ingroup loyalty, resource distribution, moral cognition, early childhood

## Abstract

The present study examined how 2- to 4-year-old preschoolers in Singapore (*N* = 202) balance fairness and ingroup loyalty in resource distribution. Specifically, we investigated whether children would enact fair distributions as defined by an equality rule, or show partiality toward their ingroup when distributing resources, and the conditions under which one distributive strategy may take precedence over the other. In Experiment 1, children distributed four different pairs of toys between two puppets. In the Group condition, one puppet was assigned to the same group as the child while the other puppet was assigned to a different group using colored stickers in the No Group condition, no group assignments were made. Children’s distributions were assessed for whether the toys were fairly (equally) distributed or unfairly (unequally) distributed in favor of either puppet. Experiment 2 was identical to the Group condition in Experiment 1, except that a third identical toy was introduced following the distribution of each toy pair. Distributions were separately assessed for whether the first two toys were fairly (equally) distributed or unfairly (unequally) distributed in favor of either puppet, and whether children distributed the third toy to the ingroup or outgroup puppet. Overall, the vast majority of children abided by an equality rule when resources were precisely enough to be shared between recipients, but distributed favorably to the ingroup member when there was limited resource availability. We found that fairness trumped ingroup loyalty except in resource distribution involving limited resources. Our results are consistent with findings from other resource distribution studies with preschoolers and similar studies measuring young infants’ expectations of distributive behaviors in third-party observations. Taken together, there is evidence suggesting stability in the development of knowledge to behavior in the subdomains of fairness and ingroup loyalty.

## Introduction

Two fundamental motivations underlie children’s decisions about resource distribution: fairness and ingroup loyalty. Fairness and ingroup loyalty represent central themes in human evolutionary history (Choi and Bowles, 2007). Fairness as a guiding principle has shaped many human communities, ranging from food-sharing practices in hunter-gatherer settlements to egalitarian sentiments in contemporary societies (Fehr et al., 2008). At the same time, ingroup loyalty is visible in many spheres of social life and encompasses biases such as favoring one’s group member in economic decisions, and in its extreme manifestation, is reflected in prejudice and gross discrimination against people who do not share the same group identity as oneself (Everett et al., 2015).

Researchers have proposed a principle-based conception of moral reasoning built on innate, domain-specific moral knowledge (Haidt and Joseph, 2004; Premack, 2007; Baillargeon et al., 2014). According to this view, fairness and ingroup loyalty are core principles in human moral cognition. In the realm of developmental research, young children are thought to simultaneously weigh fairness considerations against social obligations toward their ingroup (Killen et al., 2006; Rutland et al., 2010). Extant literature suggests that infants as young as 2 years old possess a rudimentary understanding of moral principles that dictate fair and loyal behaviors. With relation to the fairness principle, a commentary by Sommerville (2018) highlighted cumulative evidence pointing to the early development of distributive fairness, in that infants expect fair resource distributions and evaluate agents according to the fairness of their distributions. With relation to the ingroup principle, infant studies have documented third-party expectations of ingroup support, such as the obligation to help and allocate limited resources to the ingroup (Jin and Baillargeon, 2017; Bian et al., 2018).

In the present study, we looked at how 2- to 4-year-old preschoolers balance concerns about fairness and ingroup loyalty in the context of resource distribution. Specifically, we investigated whether 2- to 4-year-old preschoolers would enact fair distributions as defined by equal distributions, or show partiality toward their ingroup when distributing resources, and the conditions under which one distributive strategy may take precedence over the other. Recent studies examined young infants’ rank-ordering of fairness and ingroup loyalty in distribution scenarios where the two principles lead to opposing outcomes (e.g., Bian et al., 2018), however, these studies focus on expectations about distributive behaviors in third-party observations. Studies with older children, who can themselves participate in resource distribution, will help to shed light on the extent to which the same trends generalize from knowledge in early infancy to behavior later in development.

On the one hand, children demonstrate a strong preference for fairness. Preverbal infants expect others to act fairly (Schmidt and Sommerville, 2011; Sloane et al., 2012), and select fair distributors as social partners (Lucca et al., 2018). Sensitivities to fairness continue to strengthen over the course of development (Geraci and Surian, 2011; Sommerville et al., 2013; Deschamps et al., 2015; Ziv and Sommerville, 2016). By around 3 years of age, children react emotionally to unequal resource distributions (LoBue et al., 2011), identify egalitarian sharing as what one should do (Smith et al., 2013), allocate rewards based on an equality rule (Thomson and Jones, 2005), and negatively judge inequitable resource allocations (McCrink et al., 2010). An altruistic tendency to uphold fairness emerges at around 5 years of age, as children will protest unequal distributions of earnings from a joint effort, regardless of whether the affected individual is oneself or a third party (Rakoczy et al., 2016). Older children also enforce fairness at a cost to themselves, choosing to share their resources equally (Smith et al., 2013), sacrificing gains to punish selfish resource allocations (Jordan et al., 2014), discarding resources to avoid unequal distributions (Shaw and Olson, 2012), and endorsing resource allocations that are free of inequality, even when the inequality is beneficial to themselves (Fehr et al., 2008; Blake and McAuliffe, 2011; McAuliffe et al., 2013). Furthermore, in a study by McAuliffe and Dunham (2017), 6- to 10-year-olds proposed equal resource splits and rejected unequal offers in an ultimatum game, both when the other player was an ingroup member and when the other player was an outgroup member. Given that the enforcement of fairness norms in resource sharing was largely unaffected by group membership, this finding suggested that fairness may trump group loyalty in resource-related decisions.

On the other hand, children’s fairness preferences are heavily modulated by group membership. Young children exhibit biases favoring those of the same race (Renno and Shutts, 2015; Qian et al., 2016), same gender (Weller and Lagattuta, 2014), and who speak the same language (Kinzler et al., 2007; Pun et al., 2018). In third-party distribution tasks, 3- to 6-year-old children were found to place ingroup loyalty before fairness by distributing resources more favorably to family or friends than to strangers (Olson and Spelke, 2008; Moore, 2009), and similarly, expect others to share more with friends than with disliked peers (Paulus and Moore, 2014). Shaw (2013)’s partiality account of resource distribution further postulates that one may use resource sharing as a cue to infer the strength of social relationship between distributor and recipients. In line with this account, 4- to 9-year-olds expected a distributor to be better friends (thus stronger ingroup status) with a recipient whom the distributor had allocated a larger quantity of desirable items compared to another recipient who received a smaller allocation (Liberman and Shaw, 2017). Additionally, various studies highlight an interplay between group affiliation and fairness expectations. An aversion to behaviors that perpetuate inequality was greater when the victim belonged to the child’s ingroup than when the victim was an outgroup member (Fehr et al., 2008; Elenbaas et al., 2016); also, a social preference for fair distributors was observed only when the fair distributor was from a racial ingroup and when the disadvantaged recipient was of an outgroup race (Burns and Sommerville, 2014).

Notably, there is evidence of cross-cultural variation in children’s fairness concerns during resource distribution. A study by Blake et al. (2015) found that by the age of 9–10 years old, children in Western societies began to abide by stringent fairness criteria which led them to reject even resource inequity that was advantageous to themselves, but this developmental trend was not observed in non-Western societies, where children would only reject disadvantageous resource inequity. In a separate study, Ugandan children chose to distribute an uneven number of items unequally between two anonymous recipients, in contrast to children in the United States who would rather throw the odd item away to maintain equality, revealing yet another cross-cultural difference in fairness concerns (Paulus, 2015).

More interestingly, children’s perception of fairness appears to differ across cultures. While 4- to 6-year-old preschoolers in China preferred equal distributions over distributions that showed a consideration of recipient need (Chai and He, 2017), children in the United States prioritized recipient need. Five- to six-year-old American preschoolers gave more resources to poorer recipients than to wealthy recipients who already had plentiful resources (Paulus, 2014; Elenbaas et al., 2016; Rizzo and Killen, 2016), suggesting that their concept of fairness encompassed rectifying existing inequalities by favoring the recipient with greater need for the resource. Similarly, while an equality preference dominated African children’s distribution of spoils following a collaborative effort, children from Western societies distributed spoils from a collaborative effort unequally depending on the amount of contribution from each recipient (Schäfer et al., 2015), thereby indicating different levels of attention to merit in their notions of fairness.

The individualism-collectivism cultural distinction also contributes to the relative weight accorded to fairness versus ingroup loyalty. Fairness is classified as an “individualizing” principle that promotes the well-being of individual agents, while ingroup loyalty is classified as a “binding” principle that places collective group interests at the forefront, sometimes at the expense of those who exist outside a restricted social circle (Graham et al., 2009). In adult studies, people from Eastern countries were found to prioritize binding principles which support group interests over individualizing principles which cater to individual welfare, and rated transgressions related to ingroup loyalty as higher on moral relevance than people from Western countries (Graham et al., 2011; Kim et al., 2012).

While current research on resource distribution has primarily targeted children living in homogeneous populations, these findings do not accommodate the full range of experiences encountered by children living in more diverse populations. Singapore, the testing ground for the present study, is positioned at the cultural crossroads of the East and West, receiving strong influences from both individualistic values of fairness and collectivistic values of ingroup loyalty (Tan and Farley, 1987). The Singapore population is multi-ethnic, consisting about 74.3% Chinese, 13.4% Malay, 9.0% Indian, and 3.2% other ethnicities (Singapore Department of Statistics, 2017), with most children raised as simultaneous bilinguals proficient in English and a mother tongue. As such, there are significant deviations in Singapore’s sociocultural circumstances from her Asian counterparts, rendering generalizations based on an East–West dichotomy less likely to be germane to Singapore. For instance, the multicultural community in Singapore comprises diverse ethnic groups living in harmony, supported by social policies that enforce norms of equality, inclusivity, and intergroup camaraderie. Since these aspects of the social environment could influence the development of egalitarian and parochial motivations, the question of how children in Singapore navigate concerns about fairness and ingroup loyalty warrants investigation.

In the present study, 2- to 4-year-old preschoolers in Singapore participated in an intergroup resource distribution task. The choice of sampling 2- to 4-year-olds was motivated by their ability to provide behavioral data, even though they are younger than what has been studied in the majority of work on behavioral equality. Children in this age group in Singapore have started to attend preschool and are thus regularly exposed to the dynamics of peer interactions which foster an appreciation of fairness and ingroup loyalty; in addition, classroom play often involves sharing toys, hence these children are well-acquainted with the act of giving and receiving resources. Moreover, children from 3 years of age have been found to engage in behavioral sanctions of harm transgressions (e.g., Vaish et al., 2011), suggesting that they not only understand moral concepts but are capable of acting in ways which reflect such an understanding.

Minimal groups were assigned to children and two animal puppets using colored stickers, such that one puppet belonged to the same group as the child (*ingroup*), while the other puppet belonged to a different group (*outgroup*). This minimal group paradigm has been successfully employed in past studies: in a study that utilized shirt color as the basis of group categorization, 5-year-olds displayed ingroup favoritism on a range of behavioral measures including explicit and implicit attitudes, expectations of reciprocity, and encoding of positive information (Dunham et al., 2011).

Participants in Experiment 1 were tasked to distribute different pairs of toys between the two puppets on four test trials. Toy distribution was compared between a Group condition and a No Group condition in which no groups were assigned, and distributions were assessed for whether the toys were fairly (equally) distributed or unfairly (unequally) distributed in favor of either puppet. Experiment 2 was identical to the Group condition of Experiment 1, except that we introduced a third identical toy following the distribution of each toy pair. This two-part distribution task allowed us to determine whether children would show ingroup loyalty when given the option to distribute a single limited resource to either an ingroup or an outgroup member.

## Experiment 1

### Methods

#### Participants

Participants were 92 typically developing children (43 males; *Mean age* = 3.09 years, *SD* = 0.45, range = 2.33–4.25 years). Consent forms were distributed at local preschools and children whose parents gave consent participated in a short testing session. 82.6% of participating children were Chinese, 7.6% were Malays, 7.6% were Indians, and 2.2% were of other ethnicities. The ethnic composition of the sample is a close approximate of the overall ethnic composition in the Singapore population. An additional 20 children were tested but excluded due to failure to distribute items between puppets on at least three out of four test trials (*n* = 19) and interference from classmates (*n* = 1). Refer to **Table 1** for age distribution of final and excluded samples. The experiment was conducted in accordance with ethical guidelines and was approved by the institutional ethics review board at Nanyang Technological University.

**Table 1:** Number of children in final and excluded samples, by age, condition, and experiment.

	Experiment 1				Experiment 2	
	Group		No group			
	Final	Excluded	Final	Excluded	Final	Excluded
2 years old	23	11	15	6	40	8^#^
3 years old	22	2	27	1	59	13^+^
4 years old	1	0	4	0	11	0

*^*#*^An additional three children were excluded for non-responsiveness on third-toy distribution. ^*+*^An additional two children were excluded for non-responsiveness on third-toy distribution.*

#### Design

The experiment was a between-subjects design with two conditions. Forty-six children were assigned to the Group condition (21 males; *Mean age* = 2.94 years, *SD* = 0.35, range = 2.42–4.25 years), and another 46 children were assigned to the No Group condition (22 males; *Mean age* = 3.24 years, *SD* = 0.50, range = 2.33–4.25 years).

#### Apparatus and Materials

A puppet stage was set up by mounting a rectangular wooden frame (105 cm wide × 75 cm high × 6.25 cm thick) upright on a table. The wooden frame was attached with strong adhesive Velcro to two weighted triangular blocks that held it securely in place. A black curtain covered the opening of the frame (95 cm × 65 cm). During the experiment, a puppeteer sat at the back of the puppet stage and was concealed behind the curtain. A camcorder was discreetly positioned to take video recordings for coding purposes.

Puppets were a tiger and two identical rhinoceroses made of furry fabric, each measuring about 25 cm × 12 cm × 8 cm. The puppets emerged from behind the black curtain at marked locations – the tiger puppet appeared alone in a central spot equidistance from both sides of the stage frame, while the rhinoceros puppets appeared together, about 35 cm apart from each other, on the left and right of the stage, respectively.

**FIGURE 1:**
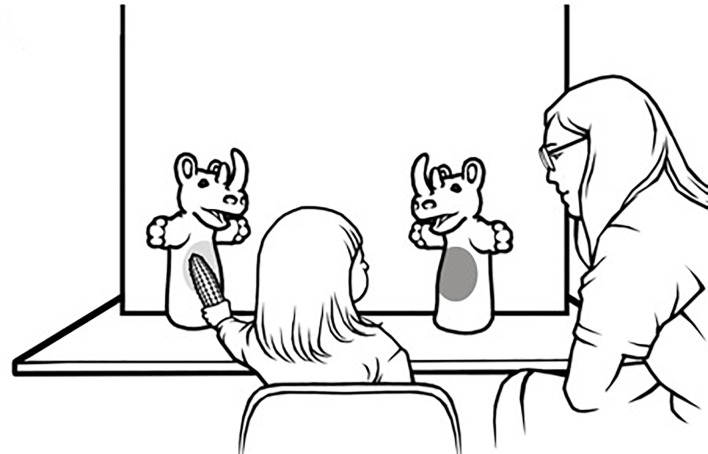
Schematic representation of experimental set-up.

The unoccupied table space in front of the stage frame was used as a platform for placing toys during distribution trials. The participating child was seated on a chair in a central position approximately 15 cm away from the puppet stage, where they could easily reach and place toys in front of the puppets. Other materials included a gray hedgehog, a blue ball, a yellow rubber duck, a small red car, two toy corns, two blocks, two toy apples, two toy rabbits and big round stickers (red and blue; 11 cm in diameter). A schematic representation of the experimental set-up is available in **Figure 1**.

#### Procedure

Children were tested one at a time at their respective preschools. Both experimenter and puppeteer were Chinese Singaporeans who spoke fluent English and Mandarin Chinese. The experiment was conducted in English with 71 participants and in Mandarin Chinese with 21 participants, depending on the child’s preferred language.

##### Warm-up phase

There were two warm-up trials for the child to practice give-and-take actions. First, the experimenter placed a toy hedgehog on the table and said, “*Look at my toy! Do you want to see it? Here you go!*” The child was then allowed to play with the toy for a few seconds before the experimenter requested, “*Now can you give the toy back to me?*” This process was repeated with a ball.

##### Familiarization phase

Next, there were two familiarization trials to familiarize the child with giving items to a puppet. A tiger puppet appeared in the center of the stage. The experimenter then introduced the puppet as Sam (if child was male) or Jessica (if child was female), took out a rubber duck, and said, “*Look, I have a toy! I want to give it away! Can you help me give the toy away?*” This process was repeated with a toy car.

##### Test phase

Following familiarization, children took part in four test trials. In the Group condition, the experimenter gave the child a red or blue sticker. Two rhinoceros puppets, introduced as Matt and Adam (if child was male), or Amy and Katie (if child was female), appeared on the left and right of the stage. One of the puppets had a red sticker affixed to its front, while the other puppet had a blue sticker. The experimenter then pointed to the puppet of the same sticker color as the child and exclaimed that the puppet’s sticker was red or blue, “*Just like yours!*” To establish group membership, the experimenter also remarked that the child and puppet were both “*on the red/blue team!*” Children’s sticker colors were randomly assigned; puppets’ sticker colors and positions (on the left or right of child) were counterbalanced across children. The No Group condition followed the same procedure except that children were not assigned any sticker color and there was no mention of them being on the same team as either puppet.

Next, the experimenter took out two toy corns and said, “*I have some corns! I want to give them away! Can you help me give the corns away?*” Once the child had distributed the corns, the experimenter put them away and repeated the instructions with three other toy pairs (blocks, apples and rabbits; in fixed order). Children had to distribute all the toys to the puppets and were not allowed to keep any toy for themselves or to discard any toy.

#### Coding

Two independent observers coded children’s distributions on each of the four test trials from video recordings. Disagreements between observers were rare, resulted from human error, and were resolved by having both observers watch the videos again. Inter-observer agreement on the final coding was 100%.

On each test trial, toy distribution was coded using the following coding scheme: a 1:1 split reflects a *fair* distribution as the two toys were divided equally between the puppets. In contrast, a 2:0 split reflects an *unfair* distribution as the child gave both toys to one of the puppets and none to the other. *Non-valid* responses include giving the toys back to the experimenter, placing the toys in between puppets, and inaction despite repeated prompts. Children who gave non-valid responses on three or more test trials were excluded from analyses (*n* = 19).

### Results

All statistical analyses were conducted with R statistical software (version 3.4.1; R Core Team, 2017). Each child had four data points, entered using a binary response term (1 = *fair*, 2 = *unfair*) for whether the child had distributed toys equally or unequally between the puppets on each test trial. Generalized linear mixed models were run on the data using the *glmer* function in R package *lme4* (Bolker et al., 2009), and child ID was fit as a random effect in all models to account for repeated measures. To test if the inclusion of predictors resulted in a significantly better model fit to the data, likelihood ratio tests (LRT) were used to compare the full model to a null model with only child ID entered as a random effect; and where predictors emerged significant, to compare the full model to a reduced model with significant predictors sequentially dropped from the full model.

Preliminary analyses confirmed that counterbalanced variables and language used for testing did not predict distribution outcomes, hence these variables were not included in subsequent analyses. The final model comprised the following predictors of interest: age in months, condition (*Group or No Group*), gender (*female or male*), and a two-way interaction between age and condition. A generalized linear mixed model yielded no significant predictor of distribution outcomes. The full model did not perform better than a null model [LRT, χ^2^(4) = 1.17, *p* = 0.88]. There was no significant effect of age (*B* = -0.36, *SE* = 0.53, *p* = 0.49), condition (*B* = 1.76, *SE* = 31.70, *p* = 0.96), gender (*B* = 1.19, *SE* = 3.00, *p* = 0.69), nor any interaction between age and condition (*B* = -0.07, *SE* = 0.97, *p* = 0.94). See **Table 2** for model output.

**Table 2:** Estimates and standard error of fixed effects in generalized linear mixed models predicting children’s distribution outcomes in Experiment 1.

	Estimate	Std. Error	*Z*-value	*P*
Intercept	1.16	17.24	0.07	0.95
Age in months	-0.36	0.53	-0.69	0.49
Condition	1.76	31.70	0.06	0.96
Gender	1.19	3.00	0.40	0.69
Age in months × Condition	-0.07	0.97	-0.08	0.94

*Reference levels for categorical variables were set at default: condition= Group, gender = female, distribution outcome = fair.*

Further analyses were conducted to examine the specific distribution pattern within each condition. Proportion of test trials with fair distribution was calculated for each child by dividing the number of trials coded as *fair*, by the total number of completed trials. All children, except three of them who completed only one or two trials, provided valid responses on all four test trials. We report the aggregate results for all children, but the exclusion of children who did not complete all four trials would not change the results.

**FIGURE 2:**
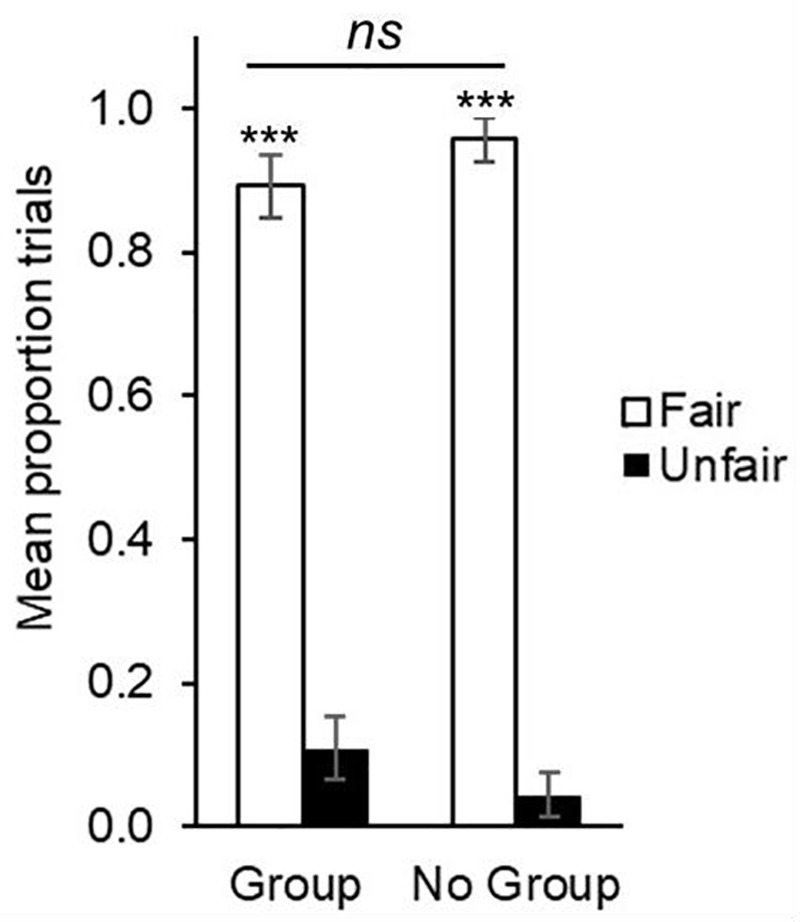
Mean proportion of trials on which children distributed two toys fairly (equally) versus unfairly (unequally) between puppets in Group and No Group conditions in Experiment 1. Error bars denote standard errors. ^∗∗∗^*p* < 0.001.

Two-tailed one-sample *t*-tests against chance (test value = 0.50) indicated that on average, children in the Group condition (*M* = 0.89, *SD* = 0.29) distributed fairly on a significantly greater proportion of trials than expected by chance, *t*(45) = 9.25, *p* < 0.001, *d* = 1.34, as did children in the No Group condition (*M* = 0.96, *SD* = 0.21), *t*(45) = 15.02, *p* < 0.001, *d* = 2.19. Results are depicted in **Figure 2**.

### Discussion

In Experiment 1, children who distributed resources in an intergroup context did not employ a different distributive strategy from children who distributed resources in the absence of a salient intergroup context. Almost all children, regardless of whether group membership had been assigned to themselves and the recipient puppets, showed a robust tendency to distribute two toys equally between the two recipients rather than favor either recipient through unequal distribution, and this tendency was consistent across multiple trials.

Because the items in the distribution task were perfectly divisible between recipients, it remains unclear how children would distribute resources between an ingroup and an outgroup member when there is a limited quantity of items that does not permit equal distribution. For example, in a study by Olson and Spelke (2008), children who were asked to distribute resources on behalf of a doll consistently enacted fair distributions when given precisely enough resources for all recipients but favored the doll’s ingroup members under conditions of resource scarcity (e.g., two items, four recipients).

A second consideration is that perhaps children did not demonstrate partiality in their distributions because the minimal groups were not sufficiently distinct and thus the puppets were not truly perceived as ingroup or outgroup members. Experiment 2 addressed this consideration using a two-part distribution task, such that children were required to distribute a third toy following the first two toys. In distributing the third toy, children had to make a forced choice between benefiting the ingroup or outgroup member. If children showed consistent ingroup favoritism on third-toy distribution, it was unlikely that the minimal group paradigm in Experiment 1 had failed to elicit clear group distinctions.

Experiment 2 was identical to the Group condition of Experiment 1, except that we introduced a third identical toy following the distribution of each toy pair. We also increased the sample size from 46 participants in the Group condition of Experiment 1 to 110 participants in Experiment 2. The rationale for increasing the sample size was that we intended for two-toy distribution in Experiment 2 to serve as a replication for the Group condition in Experiment 1, where we found close to 90% mean proportion of fair trials. We wanted to confirm those results with a larger sample that would provide greater power.

## Experiment 2

### Methods

#### Participants

One hundred and ten children (58 males; *Mean age* = 3.21 years, *SD* = 0.49, range = 2.42–4.25 years) were tested at local preschools after obtaining parental consent. 85.5% of participating children were Chinese, 3.6% were Malays, 8.2% were Indians, and 2.7% were of other ethnicities. The ethnic composition of the sample is a close approximate of the overall ethnic composition in the Singapore population. Another 21 children were tested but excluded due to failure to distribute items between puppets on at least three out of four test trials (*n* = 19) and interference from classmates or teachers (*n* = 2). Refer to **Table 1** for age distribution of final and excluded samples. The experiment was conducted in accordance with ethical guidelines and was approved by the institutional ethics review board at Nanyang Technological University.

#### Apparatus and Materials

The same puppet stage and puppets in Experiment 1 were used. Materials were identical, except that there were three instead of two of each toy (corns, blocks, apples, and rabbits).

#### Procedure

The experiment was conducted in English with 85 participants and in Mandarin Chinese with 25 participants, depending on the child’s preferred language. The procedure was identical to the Group condition in Experiment 1, except that on each test trial, after the child had distributed the first two toys, the experimenter took out an identical third toy and said, “*I found one more (corn/block/apple/rabbit)! I want to give this one away too! Can you help me give this one away?*”

#### Coding

Children’s distributions of the first two toys on each of the four test trials were observed and coded from video recordings using the same coding scheme in Experiment 1. In addition, each test trial was coded for whether the *third toy* was given to the *ingroup* or *outgroup* puppet. Non-valid responses on three or more trials resulted in exclusion from analyses (*n* = 19 for two-toy distribution; an additional *n* = 5 for third-toy distribution). Like in Experiment 1, disagreements between observers were rare, resulted from human error, and were resolved by having both observers watch the videos again. Inter-observer agreement on the final coding was 100%.

### Results

Experiment 2 followed the same analyses as Experiment 1. In addition, on third-toy distribution, each child had four data points, entered using a binary response term (1 = *ingroup*, 2 = *outgroup*) for whether the child had distributed the third toy to the ingroup or outgroup puppet on each test trial.

Preliminary analyses confirmed that counterbalanced variables and language used for testing did not predict outcomes on both two-toy and third-toy distribution, hence these variables were not included in subsequent analyses. The final model comprised the following predictors of interest: age in months and gender (*female or male*).

#### Two-Toy Distribution

A generalized linear mixed model yielded no significant predictor of two-toy distribution outcomes. The full model performed no better than a null model [LRT, χ^2^(2) = 0.02, *p* = 0.99]. There was no significant effect of age [*B* = -0.02, *SE* = 0.13, *p* = 0.89] or gender [*B* = -0.002, *SE* = 1.48, *p* = 1.00]. See **Table 3** for model output.

**Table 3:** Estimates and standard error of fixed effects in generalized linear mixed models predicting children’s distribution outcomes in Experiment 2.

	Two-toy distribution				Third-toy distribution			
	Estimate	Std. Error	*Z*-value	*P*	Estimate	Std. Error	*Z*-value	*P*
Intercept	-8.75	5.16	-1.70	0.09	0.31	1.34	0.23	0.82
Age in months	-0.02	0.13	-0.14	0.89	-0.02	0.03	-0.68	0.49
Gender	-0.002	1.48	-0.002	1.00	-0.71	0.41	-1.76	0.08

*Reference levels for categorical variables were set at default: gender = female, distribution outcome = fair (on two-toy distribution) or ingroup (on third-toy distribution).*

**FIGURE 3:**
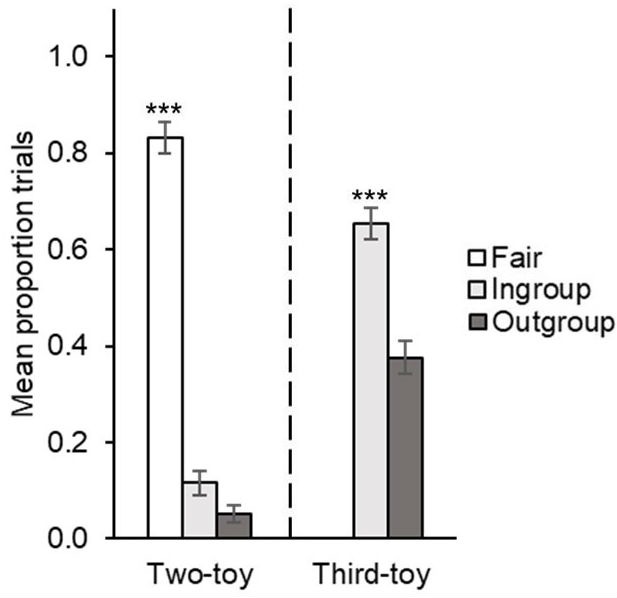
Mean proportion of trials on which children distributed fairly (equally), favored the ingroup, or favored the outgroup on two-toy distribution (on left); mean proportion of trials on which children distributed the third toy favorably to ingroup versus outgroup (on right), in Experiment 2. Error bars denote standard errors. ^∗∗∗^*p* < 0.001.

To further examine the distribution pattern, proportion of test trials with fair two-toy distribution was calculated for each child by dividing the number of trials coded as *fair*, by the total number of completed trials. All children completed all four test trials. A two-tailed one-sample *t*-test against chance (test value = 0.50) revealed that on average, children distributed fairly on a significantly greater proportion of trials (*M* = 0.83, *SD* = 0.34) than expected by chance, *t*(109) = 10.32, *p* < 0.001, *d* = 0.98. Results are depicted in **Figure 3**.

#### Third-Toy Distribution

A generalized linear mixed model found no significant predictor of third-toy distribution outcomes. The full model performed no better than a null model [LRT, χ^2^(2) = 3.62, *p* = 0.16]. There was no significant effect of age [*B* = -0.02, *SE* = 0.03, *p* = 0.49] or gender [*B* = -0.71, *SE* = 0.41, *p* = 0.08]. See **Table 3** for model output.

To further examine the distribution pattern, proportion of test trials on which the third toy was distributed to ingroup instead of outgroup puppet was calculated for each child by dividing the number of trials coded as *ingroup*, by the total number of completed trials. All children, except three of them who did not respond on one trial, completed all four test trials. We report the aggregate results for all children, but the exclusion of children who did not complete all four trials would not change the results.

A two-tailed one-sample *t*-test against chance (test value = 0.50) indicated that on average, children distributed favorably to their ingroup on a significantly greater proportion of trials (*M* = 0.65, *SD* = 0.34) than expected by chance, *t*(104) = 4.62, *p* < 0.001, *d* = 0.45. See **Figure 3** for graphical depiction of results.

### Discussion

In Experiment 2, we found that children alternated between fairness and ingroup loyalty on a two-part distribution task: they tended to be fair by distributing the first two items equally between the two recipients but exhibited ingroup loyalty by distributing the third item preferentially to the ingroup recipient. Since group membership influenced to whom children distributed the third limited resource, it is unlikely that the same minimal groups had been ineffective in creating an intergroup setting in Experiment 1. Therefore, the results in Experiment 1, which were replicated by two-toy distribution in Experiment 2, truly reflected children’s choice of fairness over ingroup loyalty when distributing an evenly divisible quantity of resources. This finding also highlights the role of resource availability as a contextual cue in guiding children’s distributive decisions.

## General Discussion

In the present study, we examined how preschoolers in Singapore weigh concerns about fairness and ingroup loyalty in an intergroup resource distribution task. Our main finding was that the vast majority of children abided by an equality rule when resources were precisely enough for two recipients but demonstrated ingroup loyalty when distribution involved a single, non-divisible resource. Overall, we found evidence that preschoolers in Singapore are predominantly fair when distributing resources and ingroup loyalty only becomes apparent under conditions of limited resource availability.

In Experiment 1, a comparison of distributive patterns between children in Group and No Group conditions suggested that group membership did not result in greater ingroup favoritism at the expense of fairness. Regardless of whether groups were assigned to the distributor and recipients, children’s distributions were largely fair (equal). One possible explanation is that resources, or the lack thereof, signal reward or punishment, such that there is resistance against unequal outcomes in resource distribution where equal outcomes are a possibility, unless the recipient demonstrates a clear lack of deservingness through inadequacies in performance or culpable conduct. There is some support for this speculation (e.g., Kenward and Dahl, 2011; Baumard et al., 2012; Surian and Franchin, 2017). Another possible explanation is that the minimal group paradigm, which relies on novel and artificial groupings such as sticker colors, had failed to elicit group identification and related intergroup processes required for ingroup loyalty to be relevant. The latter possibility was dismissed by the results obtained in Experiment 2, which similarly used minimal groups – when children had to make a forced choice between an ingroup and an outgroup member as the recipient of a limited resource, they took the course of action that benefited the ingroup member. Because Experiment 2 effectively elicited expressions of ingroup loyalty using an identical minimal group paradigm, there is evidence that the lack of group effect on resource distribution in Experiment 1 could be attributed to a robust tendency to disregard group membership when there are clear opportunities for equality, and not to an unsuccessful group manipulation.

Our findings are consistent with prior studies on children’s expectations of resource distribution. A study by Bian et al. (2018) found that 1- to 2-year-olds expected an animal puppet to distribute items equally between two other animal puppets regardless of whether the recipient was of the same species or a different species from the distributor, however, when there were just enough items for the ingroup, infants expected the distributor to exclude the different-species outgroup and give all items to the same-species ingroup. In another study, 5- to 10-year-olds expected human agents to distribute resources favorably to their own group when the groups were described to be competing over scarce resources (DeJesus et al., 2014). Based on these studies, children expect ingroup loyalty to override fairness in resource distribution involving limited resources.

Our findings are also consistent with prior studies on children’s resource distribution. In a study by Olson and Spelke (2008), children were asked to distribute resources when there were sufficient resources for all recipients and when there were insufficient resources to go around. The study found that children distributed equally regardless of the social relationship with recipients, only favoring kin and friends over strangers when resources were not enough for everyone. Unlike in the current study, however, children were told to act as proxies for a doll, such that the social relationship with recipients and distributive decisions were both established in relation to the doll, and hence the results were conceptually more reflective of children’s beliefs about the normative behaviors of others rather than their own distributive patterns. Similarly, another study found that children were more likely to favor race and gender ingroups when resources did not suffice for an equal distribution compared to when there were enough resources for every recipient (Renno and Shutts, 2015). In light of these findings, a likely explanation for the salience of ingroup loyalty under conditions of resource scarcity is offered by theories of intergroup conflict suggesting that the struggle to secure limited resources fuels competition between groups (realistic group conflict theory, Jackson, 1993). Ingroup loyalty is also thought to be linked to resource conflicts in our ancestral past as our predecessors worked in groups to obtain and protect valued resources from outgroup aggressors during a period of intergroup strife for survival (Benozio and Diesendruck, 2015).

While there is reason to expect that ingroup loyalty may be dominant in Singapore because of a collectivistic orientation, our findings on two-toy distribution suggest otherwise, echoing most of the work in Western samples where fairness trumps other types of concerns early in development (e.g., McAuliffe and Dunham, 2017). Nevertheless, it is clear from prior work (e.g., Misch et al., 2014, 2016), and from the results of third-toy distribution in Experiment 2, that children are concerned with ingroup loyalty; they simply do not manifest this concern in the context of third-party resource distribution, when resources are deemed to be sufficient for equal sharing and no additional contextual cues are provided save for group membership.

Although the present study defines fairness based on an equality rule (i.e., ensuring each recipient gets the same number of resources), this is a restrictive definition, because unequal distributions may sometimes be perceived as fair, such as when one recipient has a greater need for the resource, has worked harder to earn the resource, has rightfully won the resource from a competitive interaction, or has been assigned a greater amount of the resource through an impartial procedure. With age, children develop a nuanced perspective of what fairness entails, one that is not restricted to absolute equality but that appeals to principles related to need, merit, impartiality, norms and social justice (Schmidt et al., 2016). While findings from the current study coincide with findings from studies in other cultures, we might observe cultural differences when the definition of fairness is not constrained to a numerically equal distribution. For example, there is cross-cultural variation in the extent to which children consider work contributions and redistribution of wealth in their distributive decisions (Chai and He, 2017).

Across both experiments in the present study, recipients were identical except for group membership, which was established using superficial group markers (i.e., sticker colors). The lack of other meaningful social information about the recipients or about the nature of intergroup relations could have led children to rely more heavily on an equal distributive strategy when resources were evenly divisible. Future research should look at a wider range of distributive contexts in which ingroup loyalty may exert greater dominance over fairness. Some factors include: group dynamics (e.g., presence of intergroup conflict, relative group status), type of resource (e.g., value and function of resource), and recipient characteristics (e.g., prosociality or antisociality, work contributions). Additionally, natural group markers like speech accent or collaborative interactions could strengthen the influence of ingroup loyalty on children’s distributive decisions, in comparison to the static presentation of ingroup and outgroup members in the present study.

A final limitation of our study relates to the use of two items to represent a state of sufficiency, in that there were sufficient resources to be distributed equally among recipients, while one item was taken to represent limited resource availability. Two items can, however, still be construed as being limited in quantity, as giving both items to one recipient leaves the other recipient with none while having four items or more would alleviate such a concern. Future studies can vary the number of distributable items to convey varying degrees of resource sufficiency and scarcity, which may in turn elicit nuanced portrayals of generosity and parochial behaviors.

In summary, preschoolers in Singapore relied largely on the fairness principle to guide distributive decisions involving an evenly divisible quantity of resources but showed ingroup loyalty when distributing a limited resource. Our results converge with findings from other resource distribution studies with preschoolers and similar studies measuring young infants’ expectations of distributive behaviors in third-party observations. Taken together, there is evidence suggesting stability in the development of knowledge to behavior in the subdomains of fairness and ingroup loyalty.

## Author Contributions

PS and KL developed the study concept and design. Data collection and data analysis were performed by KL, who also drafted a first manuscript. PS and GE provided critical revisions. All authors approved the final version of the manuscript for submission.

## Conflict of Interest Statement

The authors declare that the research was conducted in the absence of any commercial or financial relationships that could be construed as a potential conflict of interest.

## References

[B1] Baillargeon R. Setoh P. Sloane S. Jin K. Bian L. (2014). “Infant social cognition: psychological and sociomoral reasoning,” in *The Cognitive Neurosciences*, 5th Edn, eds Gazzaniga M. S. Mangun G. R. (Cambridge, MA: MIT Press), 7–14.

[B2] Baumard N. Mascaro O. Chevallier C. (2012). Preschoolers are able to take merit into account when distributing goods. *Dev. Psychol.* 48 492–498. 10.1037/a0026598 22148948

[B3] Benozio A. Diesendruck G. (2015). Parochialism in preschool boys’ resource allocation. *Evol. Hum. Behav.* 36 256–264. 10.1016/j.evolhumbehav.2014.12.002

[B4] Bian L. Sloane S. Baillargeon R. (2018). Infants expect in group support to override fairness when resources are limited. *Proc. Natl. Acad. Sci. U.S.A.* 115 2705–2710. 10.1073/pnas.1719445115 29483252 PMC5856544

[B5] Blake P. R. McAuliffe K. (2011). “I had so much it didn’t seem fair”: Eight-year-olds reject two forms of inequity. *Cognition* 120 215–224. 10.1016/j.cognition.2011.04.006 21616483

[B6] Blake P. R. McAuliffe K. Callaghan T. Corbit J. Barry O. Bowie A. (2015). The ontogeny of fairness in seven societies. *Nature* 528 258–261. 10.1038/nature15703 26580018

[B7] Bolker B. M. Brooks M. E. Clark C. J. Geange S. W. Poulsen J. R. Stevens M. H. H. (2009). Generalized linear mixed models: a practical guide for ecology and evolution. *Trends Ecol. Evol.* 24 127–135. 10.1016/j.tree.2008.10.008 19185386

[B8] Burns M. P. Sommerville J. A. (2014). “I pick you”: the impact of fairness and race on infants’ selection of social partners. *Front. Psychol.* 5:93 10.3389/fpsyg.2014.00093 PMC3921677 24575069

[B9] Chai Q. He J. (2017). Chinese preschoolers’ resource allocation in the face of existing inequality under collaborative and noncollaborative contexts. *Dev. Psychol.* 53 1494–1500. 10.1037/dev0000359 28530436

[B10] Choi J. K. Bowles S. (2007). The coevolution of parochial altruism and war. *Science* 318 636–640. 10.1126/science.1144237 17962562

[B11] DeJesus J. Rhodes M. Kinzler K. (2014). Evaluations versus expectations: Children’s divergent beliefs about resource distribution. *Cogn. Sci.* 38 178–193. 10.1111/cogs.12093 24117730

[B12] Deschamps T. D. Eason A. E. Sommerville J. A. (2015). Infants associate praise and admonishment with fair and unfair individuals. *Infancy* 21 478–504. 10.1111/infa.12117 27570495 PMC4999074

[B13] Dunham Y. Baron A. S. Carey S. (2011). Consequences of “minimal” group affiliations in children. *Child Dev.* 82 793–811. 10.1111/j.1467-8624.2011.01577.x 21413937 PMC3513287

[B14] Elenbaas L. Rizzo M. T. Cooley S. Killen M. (2016). Rectifying social inequalities in a resource allocation task. *Cognition* 155 176–187. 10.1016/j.cognition.2016.07.002 27423813 PMC4983266

[B15] Everett J. A. C. Faber N. S. Crockett M. (2015). Preferences and beliefs in ingroup favoritism. *Front. Behav. Neurosci.* 9:15 10.3389/fnbeh.2015.00015 PMC4327620 25762906

[B16] Fehr E. Bernhard H. Rockenbach B. (2008). Egalitarianism in young children. *Nature* 454 1079–1083. 10.1038/nature07155 18756249

[B17] Geraci A. Surian L. (2011). The developmental roots of fairness: infants’ reactions to equal and unequal distributions of resources. *Dev. Sci.* 14 1012–1020. 10.1111/j.1467-7687.2011.01048.x 21884317

[B18] Graham J. Haidt J. Nosek B. A. (2009). Liberals and conservatives rely on different sets of moral foundations. *J. Pers. Soc. Psychol.* 96 1029–1046. 10.1037/a0015141 19379034

[B19] Graham J. Nosek B. A. Haidt J. Iyer R. Koleva S. Ditto P. H. (2011). Mapping the moral domain. *J. Pers. Soc. Psychol.* 101 366–385. 10.1037/a0021847 21244182 PMC3116962

[B20] Haidt J. Joseph C. (2004). Intuitive ethics: How innately prepared intuitions generate culturally variable virtues. *Daedalus* 133 55–66. 10.1162/0011526042365555

[B21] Jackson J. W. (1993). Realistic group conflict theory: a review and evaluation of the theoretical and empirical literature. *The Psychol. Rec.* 43 395–413.

[B22] Jin K. Baillargeon R. (2017). Infants possess an abstract expectation of ingroup support. *Proc. Natl. Acad. Sci. U.S.A.* 114 8199–8204. 10.1073/pnas.1706286114 28716902 PMC5547641

[B23] Jordan J. McAuliffe K. Warneken F. (2014). Development of in-group favoritism in children’s third-party punishment of selfishness. *Proc. Natl. Acad. Sci. U.S.A.* 111 12710–12715. 10.1073/pnas.1402280111 25136086 PMC4156744

[B24] Kenward B. Dahl M. (2011). Preschoolers distribute scarce resources according to the moral valence of recipients’ previous actions. *Dev. Psychol.* 47 1054–1064. 10.1037/a0023869 21604863

[B25] Killen M. Margie N. G. Sinno S. (2006). “Morality in the context of intergroup relationships,” in *Handbook of Moral Development*, eds Killen M. Smetana J. G. (Mahway, NJ: Lawrence Erlbaum Associates), 155–183.

[B26] Kim K. R. Kang J.-S. Yun S. (2012). Moral intuitions and political orientation: similarity and differences between Korea and the United States. *Psychol. Rep.* 111 173–185. 10.2466/17.09.21.PR0.111.4.173-185 23045859

[B27] Kinzler K. D. Dupoux E. Spelke E. S. (2007). The native language of social cognition. *Proc. Natl. Acad. Sci. U.S.A.* 104 12577–12580. 10.1073/pnas.0705345104 17640881 PMC1941511

[B28] Liberman Z. Shaw A. (2017). Children use partial resource sharing as a cue to friendship. *J. Exp. Child Psychol.* 159 96–109. 10.1016/j.jecp.2017.02.002 28285046

[B29] LoBue V. Nishida T. Chiong C. DeLoache J. S. Haidt J. (2011). When getting something good is bad: even three-year-olds react to inequality. *Soc. Dev.* 20 154–170. 10.1111/j.1467-9507.2009.00560.x

[B30] Lucca K. Pospisil J. Sommerville J. A. (2018). Fairness informs social decision making in infancy. *PLoS One* 13:e0192848 10.1371/journal.pone.0192848 PMC5812650 29444166

[B31] McAuliffe K. Blake P. R. Kim G. Wrangham R. W. Warneken F. (2013). Social influences on inequity aversion in children. *PLoS One* 8:e80966 10.1371/journal.pone.0080966 PMC3846671 24312509

[B32] McAuliffe K. Dunham Y. (2017). Fairness overrides group bias in children’s second-party punishment. *J. Exp. Psychol.* 146 485–494. 10.1037/xge0000244 28383989

[B33] McCrink K. Bloom P. Santos L. R. (2010). Children’s and adults’ judgments of equitable resource distributions. *Dev. Sci.* 13 37–45. 10.1111/j.1467-7687.2009.00859.x 20121861

[B34] Misch A. Over H. Carpenter M. (2014). Stick with your group: young children’s attitudes about group loyalty. *J. Exp. Child Psychol.* 126 19–36. 10.1016/j.jecp.2014.02.008 24842584

[B35] Misch A. Over H. Carpenter M. (2016). I won’t tell: young children show loyalty to their group by keeping group secrets. *J. Exp. Child Psychol.* 142 96–106. 10.1016/j.jecp.2015.09.016 26513328

[B36] Moore C. (2009). Fairness in children’s resource allocation depends on the recipient. *Psychol. Sci.* 20 944–948. 10.1111/j.1467-9280.2009.02378.x 19515118

[B37] Olson K. R. Spelke E. S. (2008). Foundations of cooperation in young children. *Cognition* 108 222–231. 10.1016/j.cognition.2007.12.003 18226808 PMC2481508

[B38] Paulus M. (2014). The early origins of human charity: developmental changes in preschoolers’ sharing with poor and wealthy individuals. *Front. Psychol.* 5:344 10.3389/fpsyg.2014.00344 PMC4071819 25018735

[B39] Paulus M. (2015). Children’s inequity aversion depends on culture: a cross-cultural comparison. *J. Exp. Child Psychol.* 132 240–246. 10.1016/j.jecp.2014.12.007 25626404

[B40] Paulus M. Moore C. (2014). The development of recipient-dependent sharing behavior and sharing expectations in preschool children. *Dev. Psychol.* 50 914–921. 10.1037/a0034169 23978297

[B41] Premack D. (2007). “Foundations of morality in the infant,” in *Social Brain Matters: Stances on the Neurobiology of Social Cognition*, eds Vilarroya O. Argimon F. Forn i (Amsterdam: Rodopi), 161–167.

[B42] Pun A. Ferera M. Diesendruck G. Hamlin J. K. Baron A. S. (2018). Foundations of infants’ social group evaluations. *Dev. Sci.* 21:e12586 10.1111/desc.12586 28703876

[B43] Qian M. K. Heyman G. D. Quinn P. C. Messi F. A. Fu G. Lee K. (2016). Implicit racial biases in preschool children and adults from Asia and Africa. *Child Dev.* 87 285–296. 10.1111/cdev.12442 26435128

[B44] R Core Team (2017). *R: A Language and Environment for Statistical Computing*. Vienna: R Foundation for Statistical Computing.

[B45] Rakoczy H. Kaufmann M. Lohse K. (2016). Young children understand the normative force of standards of equal resource distribution. *J. Exp. Child Psychol.* 150 396–403. 10.1016/j.jecp.2016.05.015 27329180

[B46] Renno M. P. Shutts K. (2015). Children’s social category-based giving and its correlates: expectations and preferences. *Dev. Psychol.* 51 533–543. 10.1037/a0038819 25706588

[B47] Rizzo M. T. Killen M. (2016). Children’s understanding of equity in the context of inequality. *Br. J. Dev. Psychol.* 34 569–581. 10.1111/bjdp.12150 27316464 PMC5064807

[B48] Rutland A. Killen M. Abrams D. (2010). A new social-cognitive developmental perspective on prejudice: the interplay between morality and group identity. *Perspect. Psychol. Sci.* 5 279–291. 10.1177/1745691610369468 26162160

[B49] Schäfer M. Haun D. B. Tomasello M. (2015). Fair is not fair everywhere. *Psychol. Sci.* 26 1252–1260. 10.1177/0956797615586188 26115962

[B50] Schmidt M. Svetlova M. Johe J. Tomasello M. (2016). Children’s developing understanding of legitimate reasons for allocating resources unequally. *Cogn. Dev.* 37 42–52. 10.1016/j.cogdev.2015.11.001

[B51] Schmidt M. F. H. Sommerville J. A. (2011). Fairness expectations and altruistic sharing in 15-month-old human infants. *PLoS One* 6:e23223 10.1371/journal.pone.0023223 PMC3188955 22003380

[B52] Shaw A. (2013). Beyond “to share or not to share”: the impartiality account of fairness. *Curr. Dir. Psychol. Sci.* 22 413–417. 10.1177/0963721413484467

[B53] Shaw A. Olson K. R. (2012). Children discard a resource to avoid inequity. *J. Exp. Psychol.* 141 382–395. 10.1037/a0025907 22004168

[B54] Singapore Department of Statistics (2017). *Population Trends 2017. Singapore: Ministry of Trade* & *Industry* Available at: https://www.singstat.gov.sg/-/media/files/publications/population/population2017.pdf

[B55] Sloane S. Baillargeon R. Premack D. (2012). Do infants have a sense of fairness? *Psychol. Sci.* 23 196–204. 10.1177/0956797611422072 22258431 PMC3357325

[B56] Smith C. E. Blake P. R. Harris P. L. (2013). I should but I won’t: Why young children endorse norms of fair sharing but do not follow them. *PLoS One* 8:e59510 10.1371/journal.pone.0059510 PMC3603928 23527210

[B57] Sommerville J. A. (2018). Infants’ understanding of distributive fairness as a test case for identifying the extents and limits of infants’ sociomoral cognition and behavior. *Child Dev. Perspect.* 12 141–145. 10.1111/cdep.12283 30140305 PMC6101045

[B58] Sommerville J. A. Schmidt M. F. H. Yun J. Burns M. (2013). The development of fairness expectations and prosocial behavior in the second year of life. *Infancy* 18 40–66. 10.1111/j.1532-7078.2012.00129.x

[B59] Surian L. Franchin L. (2017). Infants reason about deserving agents: a test with distributive actions. *Cogn. Dev.* 44 49–56. 10.1016/j.cogdev.2017.08.009

[B60] Tan C. T. Farley J. U. (1987). The impact of cultural patterns on cognition and intention in Singapore. *J. Consum. Res.* 13 540–544. 10.1086/209087

[B61] Thomson N. R. Jones E. F. (2005). Children’s, adolescents’, and young adults’ reward allocations to hypothetical siblings and fairness judgments: effects of actor gender, character type, and allocation pattern. *J. Psychol.* 139 349–367. 10.3200/JRLP.139.4.349-368 16097274

[B62] Vaish A. Missana M. Tomasello M. (2011). Three-year-old children intervene in third-party moral transgressions. *Br. J. Dev. Psychol.* 29 124–130. 10.1348/026151010X532888 21288257

[B63] Weller D. Lagattuta K. H. (2014). Children’s judgments about prosocial decisions and emotions: gender of the helper and recipient matters. *Child Dev.* 85 2011–2028. 10.1111/cdev.12238 24611809

[B64] Ziv T. Sommerville J. A. (2016). Developmental differences in infants’ fairness expectations from 6 to 15 months of age. *Child Dev.* 88 1930–1951. 10.1111/cdev.12674 27869290 PMC5438913

